# A Case of Bilateral Permanent Subluxation of the Lateral Meniscus

**DOI:** 10.1155/2016/5912841

**Published:** 2016-09-27

**Authors:** Jun Suganuma, Tadashi Sugiki, Yutaka Inoue

**Affiliations:** Department of Orthopaedic Surgery, Hiratsuka City Hospital, 1-19-1 Minamihara, Hiratsuka, Kanagawa 254-0065, Japan

## Abstract

We report a case of bilateral, permanent subluxation of the lateral meniscus. To our knowledge, the present case is the first reported description of bilateral irreducible anterior dislocation of the posterior segment of the lateral meniscus. This disorder is characterized by a flipped meniscus sign of the lateral meniscus on sagittal magnetic resonance images of the knee joint, with no history of trauma or locking symptoms. A detailed examination of serial magnetic resonance images of the lateral meniscus can help differentiate this condition from malformation of the lateral meniscus, that is, a double-layered meniscus. We recommend two-stage treatment for this disorder. First, the knee joint is kept in straight position for 3 weeks after the lateral meniscus is reduced to the normal position. Second, if subluxation of the lateral meniscus recurs, meniscocapsular suture is then performed. Although subluxation of the lateral meniscus without locking symptoms is rare, it is important to be familiar with this condition to diagnose and treat it correctly.

## 1. Introduction 

We present a case in which the bilateral posterior segments of the lateral menisci were dislocated anteriorly and irreducibly. To our knowledge, this is the first reported description of permanent subluxation of the lateral menisci. However, there is a previously reported case of bilateral malformation of the lateral menisci, in which the pathological characteristics resemble those of the current case [1]. The aim of this report is to differentiate permanent subluxation of the lateral meniscus from meniscal malformation, especially the double-layered lateral meniscus [2], and recommend a treatment for this pathology.

## 2. Case Report 

This case report was approved by the Institutional Review Board of our hospital. A 37-year-old housewife was referred to our knee joint clinic in January 2011 with a complaint of bilateral knee pain. In August 2010, she had been practicing a dance that involved hopping alternately on her right and left legs when she suddenly experienced severe pain in her right knee joint. She was unable to move her knee joint or walk. Before the incident, she had not engaged in athletic activities or experienced any problems with her knee joints. She consulted an orthopaedist, had a radiograph taken, and was diagnosed as having no serious problems. However, the pain and swelling of the right knee joint persisted, despite treatment with ointment and analgesics. In December 2010, she started suffering from slight left knee pain during daily activity.

**Figure 1:**
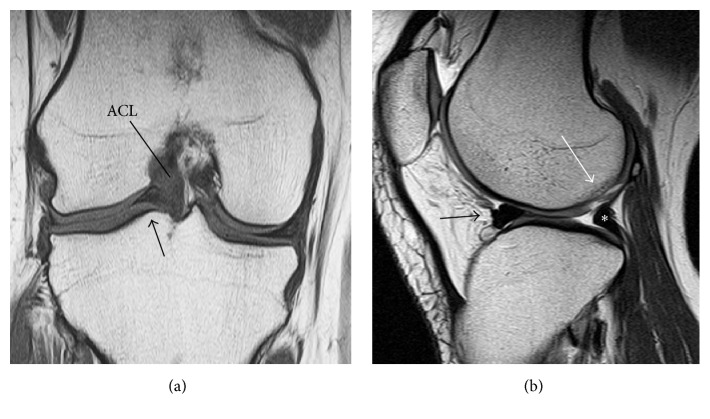
Magnetic resonance imaging of the right knee joint. (a) T1-weighted coronal image showing the posterior segment of the lateral meniscus dislocated into the intercondylar space. The arrow indicates the dislocated posterior segment, which is adjacent to the anterior cruciate ligament (ACL). (b) T2-weighted sagittal image of the lateral compartment depicting the middle segment of the lateral meniscus flipped onto the anterior segment, producing the flipped meniscus sign (black arrow). The popliteus tendon is located on the tibial plateau (asterisk) and displays a similar appearance to the posterior segment of the lateral meniscus. The white arrow indicates an osteochondral defect on the lateral femoral condyle.

The clinical examination revealed slight swelling, quadriceps muscle atrophy, and pain during the McMurray test manoeuvre for the lateral meniscus in the right knee joint; there were no other abnormal findings. The range of motion of both knee joints was 0 to 155°. Laboratory examination showed no abnormalities. On anteroposterior radiographs of both knee joints, a suspicious osteophyte was seen on the lateral tibial plateau (LTP) (Grade 1 according to the Kellgren-Lawrence grading system). On coronal views from magnetic resonance images (MRI) of both knee joints, the posterior segment of the lateral meniscus was seen in the intercondylar space, and there was only a small space for the lateral meniscus in its normal location between the articular surfaces of the lateral femoral condyle (LFC) and the LTP (Figure 1(a)). On sagittal views, a dislocated meniscus was seen on the anterior segment of the lateral meniscus, depicting the flipped meniscus sign (Figure 1(b)) [3]. The popliteus tendon ran from the LFC into the articular space between the LFC and LTP instead of running distally around the LFC and LTP. Consequently, the popliteus tendon initially looked like the posterior segment of the lateral meniscus, although the popliteus tendon was not located on the articular surfaces as closely as the posterior segment of the lateral meniscus normally is. An osteochondral defect was detected on the LFC in the right knee joint.

**Figure 2:**
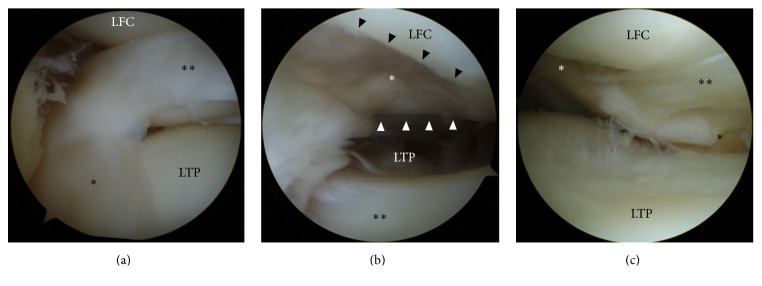
Arthroscopic findings of the right knee joint. (a) The anteriorly dislocated middle segment of the lateral meniscus (double asterisks). The asterisk indicates the anterior horn of the lateral meniscus, which is located in the normal position. (b) An osteochondral defect (asterisk) on the lateral femoral condyle (LFC). The black arrowheads indicate the anterior margin of the defect, and the white arrowheads indicate the posterior margin. The double asterisks indicate the anteriorly dislocated middle segment of the lateral meniscus. (c) The popliteus tendon (white asterisk) originating from the LFC, running on the articular surface of the lateral tibial plateau (LTP), and continuing to the popliteus muscle (black asterisk). A fibrous band (double asterisks) connects the popliteus tendon to the dislocated posterior horn of the lateral meniscus.

Arthroscopy of both knee joints was performed through anteromedial and anterolateral portals using a 30° angled arthroscope in February 2011. On arthroscopic examination of the right knee joint, the middle segment of the lateral meniscus was dislocated anteriorly on the anterior segment of the lateral meniscus (Figure 2(a)), and the posterior segment was located in the intercondylar space adjacent to the anterior cruciate ligament. An oval osteochondral defect (International Cartilage Repair Society (ICRS) Grade 4) measuring about 15 × 20 mm was detected on the LFC (Figure 2(b)). As the dislocated meniscus had adhered to tissue inside the intercondylar space, it could not be reduced to its normal position using a probe, although some of the dislocated part was barely reduced to the centre of the LTP. The popliteus tendon was running on the articular surface of the LTP and had a similar appearance to the posterior segment of the lateral meniscus (Figure 2(c)). There was a fibrous band connecting the popliteus tendon to the dislocated posterior horn of the lateral meniscus. The articular cartilage of the LTP was slightly frayed (ICRS Grade 2). There were no abnormal findings in the medial or patellofemoral compartments or in the cruciate ligaments. The arthroscopic findings of the left knee joint were almost identical to those of the right knee joint, except for the osteochondral defect of the LFC in the right knee joint.

The patient has not requested any further surgical treatment after symptoms in bilateral knee joints were alleviated with rehabilitation.

## 3. Discussion 

The pathoanatomic features of this case are similar to those in recurrent subluxation of the lateral meniscus (RSLM) [4]. In most cases of RSLM, the first locking symptoms occur with severe pain when patients extend their knee joint from deep flexion [4–6]; the youngest reported age at which the first locking symptoms have occurred is 6 years [4]. Both disorders show subluxation of the lateral meniscus without tears or anomalies. The only difference between RSLM and this case is that the lateral meniscus is subluxated repeatedly in the former [4, 6], while the subluxated lateral meniscus cannot be reduced in the latter. Therefore, we have named this disorder permanent subluxation of the lateral meniscus (PSLM).

The cause of dislocation of the posterior segment of the lateral meniscus without tears or anomalies or instability of the knee joint has yet to be elucidated. However, the onset of locking symptoms seems to be related to several factors, including insufficiency of the popliteomeniscal fascicles [7] and internal rotation of the knee joint [4]. The developmental mechanism of PSLM seems to be even more complicated and appears to be related to both congenital and environmental factors, as the dislocated lateral meniscus needs to be adhered to the surrounding tissue.

As the present case involved irreducible anterior dislocation of the posterior segment of the lateral menisci in bilateral knee joints without history of evident trauma or locking symptoms, these subluxations must have occurred in an early stage of the patient's life. In embryological studies of the human knee joint, knee extension decreases gradually from 33 weeks owing to the lack of space in the uterus, and the knee joints are kept in a deeply flexed position from 38 weeks [8]. Therefore, late gestation can be a vulnerable period for the lateral menisci of foetuses because locking symptoms of RSLM usually occur related to deep flexion of the knee joint [4–6]. It seems that anterior dislocation of the posterior segment of the lateral meniscus can occur when risk factors such as insufficiency of the popliteomeniscal fascicles and internal rotation of the knee joint are present; this dislocation could lead to irreducible subluxation of the lateral meniscus if the dislocated meniscus is not reduced spontaneously during extension of the knee joint.

There is a previously reported case in which the pathological characteristics had a distinct resemblance to the present case. Fujikawa et al. reported the case of a 37-year-old man with bilateral lateral meniscal malformation consisting of a duplicated anterior horn and the absence of the lateral portion of the menisci [1]. They did not think that the condition was caused by a displaced bucket-handle tear, because they could not move the duplicated smooth-surfaced menisci. They also did not observe mobile unstable fragments or a ragged free margin, which are expected in displaced bucket-handle tears. The significant difference between the previous case and our present case is that their case involved marked osteoarthritic changes in the LFC on the MRI; this osteoarthritis was probably due to the higher level of activity of their patient.

There have been multiple reports on meniscal malformations, which are categorized into three groups: hyperplastic (discoid, double-layered [2], abnormal band [9], accessory [10], and ring-shaped [11]), hypoplastic (absence [12], partial deficiency [13], and separation [14]), and insertional anomalies. It does not seem difficult to differentiate PSLM from meniscal malformations, because PSLM does not involve any malformation. However, PSLM can be seen as a combination of two malformations: double-layered and partially deficient meniscus. A meniscus whose posterior segment is dislocated anteriorly can look like a double-layered anterior horn. Furthermore, the popliteus tendon in PSLM looks like the posterior segment of the lateral meniscus, as the popliteus tendon runs on the articular surface of the LTP. Consequently, only the middle segment of the lateral meniscus might look deficient. However, a detailed examination of serial MRI of the lateral meniscus and the popliteus tendon reveals that there is neither duplication nor deficiency in the lateral meniscus, and the structure that appears to be the posterior segment of the lateral meniscus is actually the popliteus tendon. Arthroscopy revealed a fibrous band that connected the popliteus tendon to the posterior horn of the lateral meniscus (Figure 2(c)). As the posterior horn of the lateral meniscus was dislocated anteriorly, the popliteus tendon might be pulled anteriorly onto the articular surface of the LTP by the fibrous band, which was considered to be the posteroinferior (or third) popliteomeniscal fascicle [15, 16].

The reason why we did not perform any interventions for the subluxated lateral menisci during arthroscopic examination is that the patient declined intensive treatment, because she was busy with child rearing and her job at that time. However, if the patient had wanted to undergo thorough treatment, we would have released the adhesion between the lateral meniscus and tissue inside the intercondylar space and reduced the subluxated meniscus to the normal position after making sure that the size of the lateral meniscus is large enough. Based on our experience with other patients in whom the lateral meniscus had been kept subluxated for more than 1 month and had then been reduced to the normal position arthroscopically, such a meniscus is liable to undergo redislocation to the same position during even slight flexion of the knee joint. To break this predisposition, we recommend two-stage treatment. First, the knee joint is kept in a straight position with a plaster cast for 3 weeks after reduction of the lateral meniscus to the normal position. The patient is then encouraged to walk with full weight bearing on the knee joint after the correct position of the lateral meniscus is verified on MRI. Reposition of the posterior segment of the lateral meniscus can push the popliteus tendon off the LTP to the normal position, which makes meniscocapsular suture [5, 6] possible. Active flexion exercise of the knee joint is started after the plaster cast is removed. Second, if subluxation of the lateral meniscus recurs, immediate repositioning of the meniscus and meniscocapsular suture, which would be much easier compared to at first repositioning of the meniscus, are performed simultaneously.

As PSLM seems to remain clinically silent until the occurrence of an osteochondral fracture or osteoarthritis caused by the loss of meniscal function, there would be little chance of detecting PSLM while patients are young. However, if a flipped meniscus sign of the lateral meniscus is detected on MRI in a knee joint without a history of trauma or locking symptoms, PSLM should be considered.

## 4. Conclusion 

Permanent subluxation of the lateral meniscus without tears or anomalies or a history of locking symptoms is a rare disorder. We recommend two-stage treatment for this pathology. First, the knee joint is kept straight for 3 weeks after reduction of the lateral meniscus to the normal position. Second, if subluxation of the lateral meniscus subsequently recurs, meniscocapsular suture is performed. We believe that this may pose a challenge clinically and radiologically; hence, preliminary knowledge of this condition is important.
